# Staged procedures for prevention of spinal cord ischemia in endovascular aortic surgery

**DOI:** 10.1007/s00772-018-0410-z

**Published:** 2018-07-02

**Authors:** F. Heidemann, N. Tsilimparis, F. Rohlffs, E. S. Debus, A. Larena-Avellaneda, S. Wipper, T. Kölbel

**Affiliations:** German Aortic Center Hamburg, Department and Outpatient Clinic for Vascular Medicine, University Heart Center, University Hospital Hamburg-Eppendorf, Martinistraße 52, 20246 Hamburg, Germany

**Keywords:** Spinal ischemia, Cerebrospinal fluid drainage, Staging procedure, Thoracic endovascular repair, Thoracoabdominal aortic aneurysm, Spinale Ischämie, Liquordrainage, Staging-Verfahren, Endovaskuläre Therapie, Thorakoabdominelles Aortenaneurysma

## Abstract

**Background:**

Spinal cord ischemia with development of paraplegia is the most relevant complication of thoracoabdominal aortic surgery caused by compromising the segmental arteries. To prevent this devastating complication in endovascular aortic surgery, staging procedures have been developed to reinforce collateral blood flood to the spinal cord.

**Results:**

In patients with a medium to high risk for spinal cord ischemia, staged aortic repair is recommended. The classical staged repair is the two-step repair with delayed implantation of the aortic stent grafts. Additionally, more recent methods for short-term salvage of segmental artery perfusion by leaving an endoleak have been developed. Perfusion branches, delayed bridging stents as well as the open branch technique are among these methods. The latest option of staged repair is minimally invasive segmental artery embolization.

**Conclusion:**

Besides the nonsurgical options for monitoring and therapy of spinal cord ischemia, various staging procedures are available, which can be implemented depending on the patient and the aortic anatomy. Evidence that underlines staged repair for endovascular treatment of thoracoabdominal aortic pathologies is mostly based on retrospective studies.

## Zusammenfassung

**Hintergrund:**

Die spinale Ischämie mit klinischer Manifestation eines kompletten Querschnittsyndroms ist eine der relevantesten Komplikationen der thorakoabdominellen Aortenchirurgie und wird perioperativ v. a. durch die Unterbindung oder Überstentung rückenmarkversorgender Segmentarterien verursacht. Zur Prävention dieser schwerwiegenden Komplikation haben sich spezielle Staging-Verfahren in der endovaskulären Aortentherapie entwickelt, die das Ziel haben, die operative Versorgung des thorakoabdominellen Aortenaneurysmas (TAAA) in mehrere Eingriffe zu unterteilen, um die Entwicklung von Kollateralkreisläufen zur Sicherung der spinalen Perfusion zu stärken.

**Ergebnisse:**

Zu den Staging-Verfahren bei erhöhtem Risiko für eine spinale Ischämie in der endovaskulären Aortentherapie zählt als klassische Methode das zweizeitige Vorgehen mit zeitversetztem Einbringen der aortalen Stentgrafts. Daneben haben sich neuere Verfahren zum kurzfristigen partiellen Erhalt der Segmentarterienperfusion durch ein geplantes Endoleak entwickelt. Hierzu zählen die Perfusionsbranches, verzögerte Bridging-Stents und die „open branch technique“. Bei diesen mehrzeitigen Verfahren werden die aortalen Stentgrafts eingebracht und die entsprechend offen belassenen Branches in einem Sekundäreingriff mit oder ohne vorheriger Testokklusion komplettiert. Die neueste Methode unter den Staging-Verfahren ist die minimal-invasive Segmentarterienembolisation.

**Schlussfolgerung:**

Neben den konservativen Methoden zur Überwachung und Therapie der spinalen Ischämie stehen mehrere Staging-Verfahren zur Verfügung, die in Abhängigkeit von Patient und Anatomie angewandt werden können. Es besteht eine Evidenz, die das mehrzeitige Vorgehen zur endovaskulären Therapie von thorakoabdominellen Aortenpathologien stützen, diese basiert aktuell noch im Wesentlichen auf retrospektive Studien.

## Introduction

Spinal cord ischemia (SCI) involving its most extreme clinical manifestation of paraplegia with loss of bowel and bladder control is one of the major complications of both open and endovascular aortic surgery. It is caused either by open aortic repair or endovascular stenting of arteries relevant to the spinal cord in combination with other risk factors, such as perioperative hypotension and anemia. Patients with long segment thoracic and thoracoabdominal aortic pathologies are particularly at risk. Due to its clinical relevance, special monitoring and staging procedures have been developed for the prevention of SCI.

This article provides an overview of the fundamentals of SCI and the methods for prevention, in particular using staging methods in endovascular aortic therapy.

## Background

An SCI causes reduced blood flow to the spinal cord, the part of the central nervous system located in the spinal canal, which is enclosed by meningeal sheaths and connected to the peripheral nervous system via spinal nerves. The spinal cord is surrounded by cerebrospinal fluid (CSF), which among other things provides mechanical protection for the spinal cord and brain. Histologically, the spinal cord is made up of gray matter with its nerve cell bodies and white matter with axons. The gray matter, which appears butterfly-shaped in cross-section, consists of the anterior horn, an area largely responsible for the innervation of skeletal muscles, and the posterior horn, which mainly transmits sensory information.

The clinical severity of SCI is subject to considerable variation. Depending on the extent of the spinal cord area affected this can result in paraparesis or paraplegia, sensory deficits, ataxia and autonomic dysfunction involving impaired bowel and bladder function. These symptoms may be transient or permanent.

## Spinal cord perfusion

**Fig. 1:**
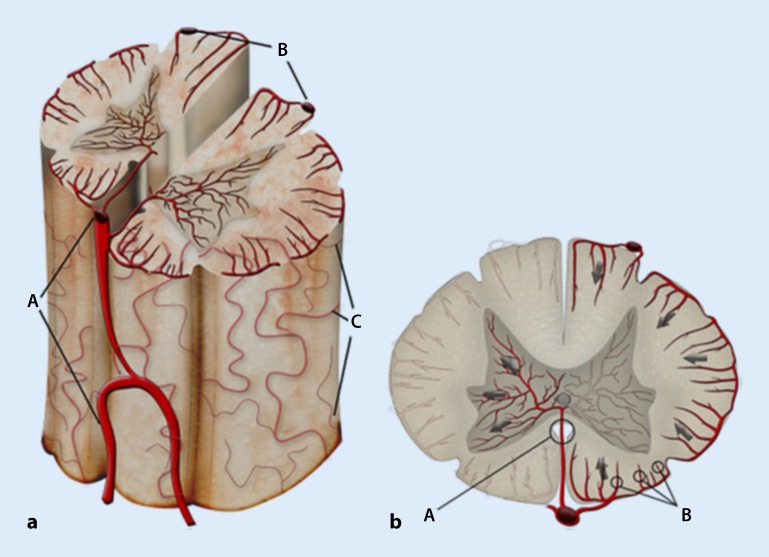
**a** Blood supply to the spinal cord via the anterior spinal artery (*A*) and two posterolateral spinal arteries (*B*), which are interconnected by anastomoses of the pial network (*C*). **b** The intrinsic arteries of the spinal cord. Left (central system): a sulcal artery (*A*), which originates from the anterior spinal artery, penetrates the spinal cord and feeds the gray matter. Right (peripheral system): this system is made up of numerous Rami perforantes (*B*) that originate from the pial network and supply the white matter (from [15], with the kind permission of Elsevier. This content is not part of the Open Access License)

The spinal cord is supplied by a network-like vascular system with afferent vessels originating in the anterior spinal artery and two posterolateral arteries that are connected longitudinally. In the cervical region, these arteries originate in the vertebral artery, while in the thoracic and lumbar region, they receive segmental supply from the intercostal, lumbar and pelvic arteries. The intrinsic system of spinal cord perfusion is divided into a central (centrifugal) system and a peripheral system (centripetal; Fig. 1). The central system comprises the sulcal arteries that originate in the anterior spinal artery and supply the gray matter with the alpha-motor neurons. The peripheral system consists of numerous small Rami perforantes that originate in the pial network and supply the white matter. The pial network covers the entire length of the spinal cord and forms strong anastomoses between the anterior and posterolateral longitudinal arteries.

There are three concepts of spinal perfusion in the context of surgical aortic repair.

### The traditional (Adamkiewicz) concept

At the end of the nineteenth century, the Polish pathologist Albert Adamkiewicz postulated that the most important artery for thoracolumbar spinal cord perfusion, the great anterior radiculomedullary artery (also known today as the artery of Adamkiewicz), originated in the region of the thoracic aorta, generally between the 9th and 12th intercostal arteries. According to the traditional concept, spinal cord integrity in the thoracic region depends largely on this predominant artery.

### The collateral network concept

The collateral network concept for spinal perfusion was developed in the early 2000s [1] and is based on the knowledge that spinal cord perfusion can be ensured by intraspinal and paraspinal arteries in the case of occlusion of segmental arteries. According to this concept, spinal circulation consists of three compartments that communicate longitudinally and horizontally, the intraspinal, the paraspinal intramuscular, and the extramuscular compartment [2]. In the case of segmental artery occlusion, these compartments are mobilized and angiogenesis is stimulated. This paraspinal and intraspinal network receives its blood supply from the various regions corresponding to the territories concept and is essentially able to compensate acute and chronic hypoperfusion of the spinal cord during open and endovascular aortic surgery.

### Vascular territories concept

This concept divides spinal cord perfusion into four feeding arterial territories (Infobox 1): the cervical arteries (in particular the vertebral artery), the intercostal arteries, the lumbar arteries, and the hypogastric arteries (internal iliac artery). Czerny et al. [3] demonstrated that extensive stenting of intercostal arteries alone was not associated with spinal ischemia but that simultaneous stenting of two territories, particularly in conjunction with prolonged intraoperative hypotension, caused a relative increase in spinal ischemia. This concept was supported by Eagelton et al. [4], among others. In their study on 1251 patients, they showed that the patency of hypogastric arteries and the left subclavian artery is relevantly associated with the avoidance of spinal ischemia in endovascular aortic aneurysm repair.

#### Infobox 1 The four territories of spinal cord perfusion

1. Supra-aortic: cervical arteries (in particular the vertebral artery)
2. Thoracic aorta: intercostal arteries
3. Abdominal aorta: lumbar arteries
4. Pelvic: internal iliac artery

The three concepts of spinal cord perfusion presented here should be seen as complementary rather than competing concepts, since they are mutually complementary and can stand side by side. Thus, while the distal thoracic segmental arteries are important branches in spinal cord blood supply, the collateral network is able to compensate for occlusions; however, the likelihood of SCI increases if at least two territories of spinal cord perfusion are compromised.

## Pathophysiology of spinal ischemia

The placement of a stent graft in the aorta can cause a drop in spinal blood flow volume in the corresponding spinal cord region. This reduction in spinal cord perfusion causes a direct cytotoxic reaction within a short space of time. In addition, edema formation occurs, accompanied by the development of spinal cord compartment syndrome and a rise in intraspinal pressure due to cytotoxic processes and in the context of reperfusion, e. g., if the mean arterial pressure (MAP) increases or hypotensive phases are normalized. The rise in intraspinal pressure in turn causes direct cell damage and a reduction in spinal perfusion.

### Infobox 2

The following formula can be used to calculate the spinal perfusion pressure relevant in the monitoring and treatment of SCI:

Spinal cord perfusion pressure (SCPP) *=*

mean arterial pressure (MAP) – cerebrospinal fluid pressure (CSFP)

## Methods to monitor and prevent spinal cord ischemia

According to the formula in Infobox 2, hypotensive phases (= MAP) as well as increased intraspinal pressure (= CSFP) have a negative effect on spinal cord perfusion pressure (SCPP). As such, blood pressure and CSFP are the adjusting screws in the conservative monitoring and treatment of spinal ischemia. Blood pressure management includes avoiding intraoperative and postoperative hypotensive phases in general, as well as maintaining MAP following segmental artery occlusion, thereby ensuring sufficient perfusion via the collateral network. At the authors’ hospital, a MAP value of 80–90 mm Hg is set for at least 48 h.

Drainage of CSF is the most frequently used method today to manage CSFP. The placement of a CSF drain should be carried out according to the risk for SCI, since this procedure is itself associated with relevant complications. Drainage of CSF can be used as a preventive measure or as a secondary measure if SCI is suspected postoperatively. At the authors’ hospital, prophylactic CSF drainage is generally used if at least two territories of spinal cord perfusion according to the vascular territories concept (see above) are impaired and cannot be reopened by means of revascularization measures (e. g., carotid artery–subclavian artery bypass).

In addition to these two important approaches, there are other factors that need to be monitored and optimized in the perioperative setting. An adequate level of central venous oxygen saturation (ScvO_2_) of ≥70%, as well as an intraoperative central venous pressure (CVP) of ≤10 mm Hg, should be aimed for. A hemoglobin level of ≥8 g/dl is desired, and intraprocedural blood loss should be kept to a minimum. The use of a cell salvage system offers the possibility to retransfuse lost autologous blood. Once a procedure has been completed, attempts should be made to extubate the patient as soon as possible in order to ascertain the postoperative neurological status. This should be monitored postoperatively at 2‑h intervals. In the case of clinically asymptomatic patients, all measures to monitor and prevent SCI should be carried out for 48 h.

## Revascularization procedures

**Fig. 2:**
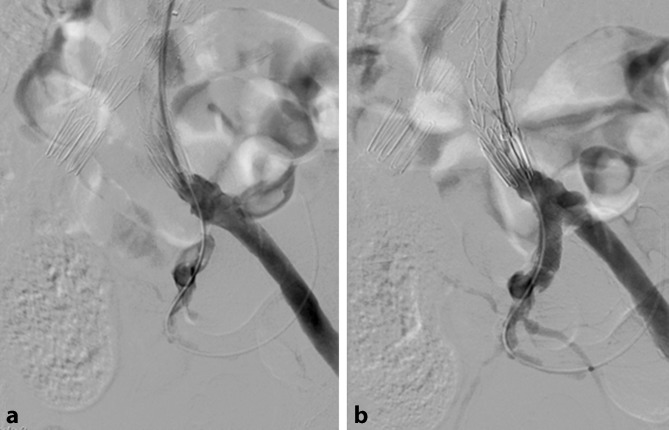
**a** A patient with fenestrated repair of a thoracoabdominal aortic aneurysm and high-grade stenosis of the left internal iliac artery. Following placement of the fenestrated stent graft, the left internal iliac artery was catheterized to reduce the risk of spinal cord ischemia. **b** Control angiography following successful placement of a short balloon-expandable stent in the left internal iliac artery to improve circulation to the pelvic territory

The vascular territories concept offers surgical options in addition to conservative methods to prevent SCI. For example, in the case of relevant stenosis or occlusion of the internal iliac artery, revascularization can be performed, e. g., using stents, either in a preliminary session or in the same session as the planned thoracoabdominal aorta (TAA) or thoracoabdominal aortic aneurysm (TAAA) repair (Fig. 2). Likewise, if stenting is planned or if there is existing stenosis of the left subclavian artery, a carotid-subclavian bypass can be performed either prior to or at the same time as the endovascular procedure. In the acute setting, i. e., in the case of symptoms or rupture, the revascularization methods mentioned above are secondary, that is to say, they can be carried out as a follow-up procedure if suspected SCI symptoms do not resolve in response to conservative methods.

## Staging procedures in endovascular aortic surgery

A crucial aspect of SCI prevention in the context of endovascular repair is the staging procedure or staged approach. The aim of the staged approach in the complete exclusion of aortic pathology is to reduce SCI risk by preconditioning the spinal cord through the development of collateral networks for spinal cord perfusion. Moreover, reducing the complexity of the procedure reduces the risk of relevant intraoperative blood loss and perioperative hypotension, thereby lowering the risk of SCI.

The following comprise the staging procedures for endovascular aortic repair:

- Classical two-step approach
- Perfusion branches
- Delayed bridging stents and test occlusion
- Segmental artery embolization

## Classical two-step approach

The classical two-step approach can be used in both open and endovascular aortic surgery. Complete exclusion of the aortic pathology is performed in two surgical procedures, with only part of the affected aorta being repaired or stented in the first procedure. This offers the option, for example in type II or III TAAA, to place only a thoracic stent graft in the first step, while a branched stent graft can be placed in the follow-up procedure for the abdominal aorta.

In a retrospective analysis of 90 patients who underwent open aortic repair for type II TAAA, Etz et al. [5] demonstrated a significant reduction in the paraplegia rate in favor of the two-step approach compared with the single-stage approach (0% vs. 15%). A comparison carried out by O’Callaghan et al. [6] of single stage and multistage endovascular treatment in 87 Crawford type II TAAA patients revealed a significant reduction in both the incidence and severity of SCI with the two-stage approach (37.5% vs. 11.1%; *p* = 0.03).

## Perfusion branches

**Fig. 3:**
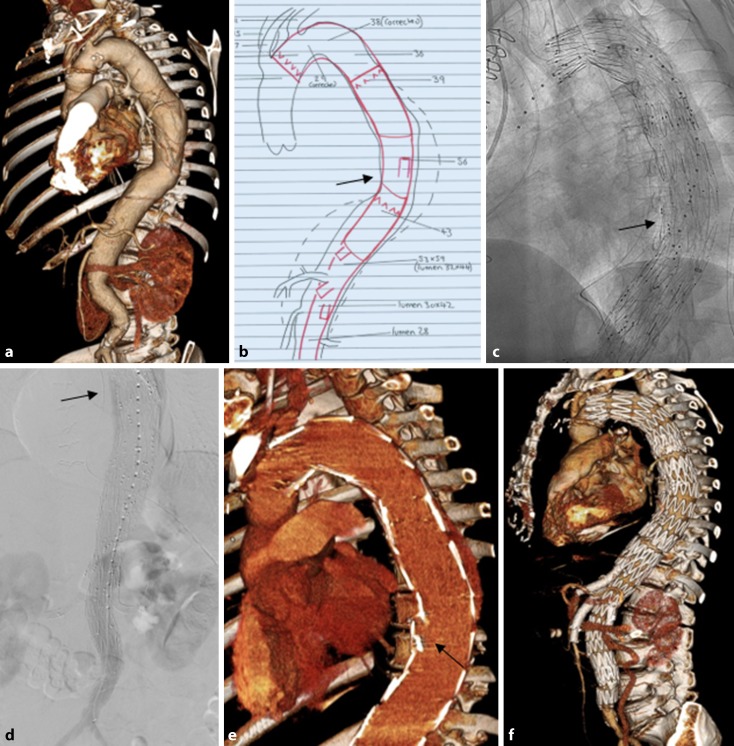
A female patient with a thoracoabdominal aortic aneurysm (**a**) treated using a four-vessel branched stent graft with a perfusion branch (*arrow *in **b**). The perfusion branch (*arrow*) following graft placement (***c***) and aneurysm perfusion (*arrow*) on late final angiography (**d**). Follow-up computed tomography angiography shows the open perfusion branch (*arrow* in **e**) and complete repair (**f**)

Perfusion branches (Fig. 3) are branches that are integrated in patient-specific, custom-made endografts and which serve to maintain a temporary endoleak in order to perfuse the aneurysm, and hence also segmental arteries. These custom-made devices with sac perfusion branches are inserted in an initial procedure and all target vessels connected accordingly, while the perfusion branch remains open. Then, in a second procedure that is often carried out with the patient under local anesthesia, the perfusion branches are closed to achieve complete aneurysmal exclusion. This second procedure can be carried out approximately 7–14 days following the initial intervention.

This method was described in 2011 by Lioupis et al. [7]. As two of the first working groups, Harrison et al. [8] with 10 patients and Jayia et al. [9] with 25 patients showed the feasibility and benefit of this staging procedure using perfusion branches.

## Delayed bridging stents, the open branch technique, and test occlusion

The delayed bridging stent method is similar to the perfusion branch technique. A branched graft is placed in a preliminary intervention, whereby a target vessel, usually the celiac artery, is not connected in order to maintain a temporary endoleak for the purposes of spinal cord perfusion. In the follow-up procedure, simultaneously to the sac perfusion branches, the procedure is then completed by connecting the side branch to the target vessel.

**Fig. 4:**
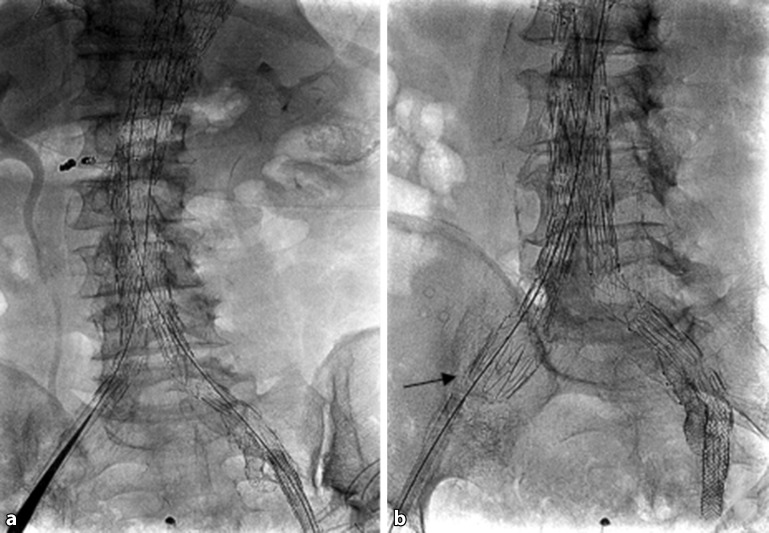
**a** Four-fenestration endovascular aortic repair without left iliac extension. **b** Completion achieved by inserting the right iliac leg (*arrow*)

In their retrospective comparative study, Kasprzak et al. [10] investigated the concept of temporary aneurysm sac perfusion (TASP) using delayed bridging stents in 83 patients and were able to demonstrate both feasibility and a reduction in SCI rate. Another variant of TASP is delayed iliac completion in the case of pathologies extending to the aortic bifurcation or iliac vessels. Here, the fenestrated or branched stent graft is introduced in a primary intervention and all target vessels connected, without, however, completing iliac supply. This leaves a type Ib endoleak for the temporary perfusion of the lumbar arteries; this endoleak is then excluded by ipsilateral or contralateral extension. The second intervention can also be performed with the patient under local anesthesia 7–14 days following the initial procedure (Fig. 4).

The open branch technique represents a technical alternative and add-on to TASP [11]. The branched stent is deployed in an initial procedure and an uncovered stent is used to connect to one of the target vessels in order to create an endoleak for aneurysm perfusion. A covered stent is then used in the follow-up procedure to achieve complete exclusion. In the authors’ view, the advantage here compared with delayed bridging stents is the lower risk of stent graft dislocation, as well as a reduced risk of target vessel occlusion. All the procedures mentioned here, from sac perfusion branches to the open branch technique, can be performed in a secondary procedure including test occlusion. This involves balloon occlusion of the hitherto unconnected branch for approximately 10–15 min. This enables the neurological status of the legs to be assessed “live” in the awake patient. If the patient remains free of neurological symptoms, the procedure can be completed accordingly. Intraoperative monitoring of motor function of the extremities is also possible in the case of general anesthesia by using motor evoked potentials.

## Segmental artery embolization

**Fig. 5:**
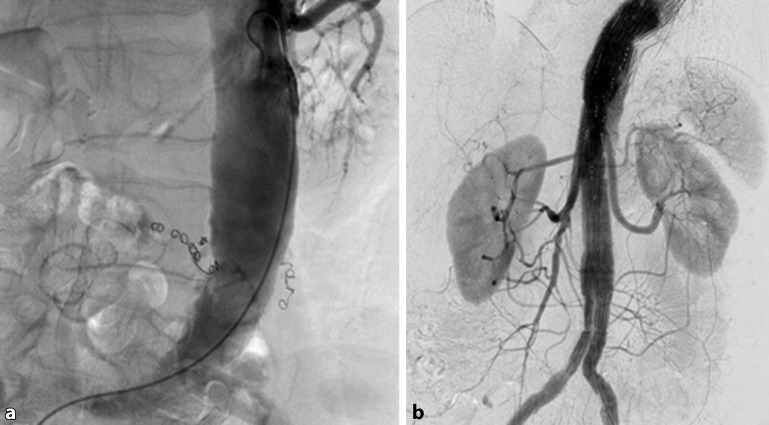
Minimally invasive segmental artery coil embolization involving coiling of two segmental arteries and the inferior mesenteric artery (**a**) prior to branched endovascular repair (**b**) of a type II TAAA according to Crawford (from [14], with kind permission from Elsevier. This content is not part of the Open Access License)

Minimally invasive segmental artery coil embolization (MISACE) is a method first published in 2014 [12, 13] in which segmental arteries are selectively embolized and thus occluded in a preliminary, minimally invasive procedure in order to mobilize arterial collateral circulation and promote angiogenesis for spinal cord perfusion (Fig. 5).

In 2015 Etz et al. [14] published the first case report on this method in two patients with Crawford type II and III TAAA. Segmental artery embolization was performed with the patient under local anesthesia in a first intervention. One patient underwent open TAA repair 4 weeks following embolization, while the second patient underwent endovascular repair after 8 weeks. Neither patient was affected by SCI and both remained free of neurological abnormalities at 1‑year follow-up. Thus, segmental artery coil embolization represents a promising novel treatment approach for the prevention of SCI prior to thoracic and thoracoabdominal aortic repair.

## Conclusion

The endovascular staging procedures described here appear to be beneficial in the prevention of SCI. Having said that, the current evidence for these staging procedures in the endovascular treatment of TAA and TAAA is still largely based on retrospective studies and case series, as well as indirectly on large studies on open aortic repair. Despite the advantages of staging procedures, however, one should not ignore the risk of rupture during the interval between procedures. Thus, the decision for or against, as well as the choice of staging procedure and the interval between interventions, always needs to be made on a case by case basis taking into account the risk-benefit ratio.

## Practical conclusion

- Staging procedures to prevent SCI can be beneficial in interventions carrying medium or high risk for impaired spinal cord circulation.
- The aim of staging procedures is to reduce the risk of SCI by creating a collateral network for spinal cord perfusion.
- Revascularization should be considered prior to endovascular treatment for stenosis or occlusion of the internal iliac artery, and a carotid-subclavian bypass should be considered prior to left subclavian artery stenting.
- In addition to the classical two-stage procedure, perfusion branches, delayed bridging stents, and the open branch technique with or without test occlusion are available as staging procedures.
- Segmental artery coil embolization is the newest approach among the staging procedures to prevent SCI.

## References

[1] Jacobs MJ (2002). Spinal cord blood supply in patients with thoracoabdominal aortic aneurysms. J Vasc Surg.

[2] Griepp RB, Griepp EB (2007). Spinal cord perfusion and protection during descending thoracic and thoracoabdominal aortic surgery: the collateral network concept. Ann Thorac Surg.

[3] Czerny M (2012). Mechanisms of symptomatic spinal cord ischemia after TEVAR: insights from the European Registry of Endovascular Aortic Repair Complications (EuREC). J Endovasc Ther.

[4] Eagleton MJ (2014). Hypogastric and subclavian artery patency affects onset and recovery of spinal cord ischemia associated with aortic endografting. J Vasc Surg.

[5] Etz CD (2010). Staged repair significantly reduces paraplegia rate after extensive thoracoabdominal aortic aneurysm repair. J Thorac Cardiovasc Surg.

[6] O’Callaghan A, Mastracci TM, Eagleton MJ (2015). Staged endovascular repair of thoracoabdominal aortic aneurysms limits incidence and severity of spinal cord ischemia. J Vasc Surg.

[7] Lioupis C (2011). Paraplegia prevention branches: a new adjunct for preventing or treating spinal cord injury after endovascular repair of thoracoabdominal aneurysms. J Vasc Surg.

[8] Harrison SC (2012). Elective sac perfusion to reduce the risk of neurologic events following endovascular repair of thoracoabdominal aneurysms. J Vasc Surg.

[9] Jayia P (2015). Temporary perfusion branches to decrease spinal cord ischemia in the endovascular treatment of thoraco-abdominal aortic aneurysms: based on a presentation at the 2013 VEITH Symposium, november 19–23, 2013 (New York, NY, USA). Aorta (Stamford).

[10] Kasprzak PM (2014). Editor’s choice – Temporary aneurysm sac perfusion as an adjunct for prevention of spinal cord ischemia after branched endovascular repair of thoracoabdominal aneurysms. Eur J Vasc Endovasc Surg.

[11] Mangialardi N (2016). The “Open Branch” technique: a new way to prevent paraplegia after total endovascular repair of thoracoabdominal aneurysm. Catheter Cardiovasc Interv.

[12] Geisbusch S (2014). Endovascular coil embolization of segmental arteries prevents paraplegia after subsequent thoracoabdominal aneurysm repair: an experimental model. J Thorac Cardiovasc Surg.

[13] Luehr M (2014). Minimally invasive segmental artery coil embolization for preconditioning of the spinal cord collateral network before one-stage descending and thoracoabdominal aneurysm repair. Innovations (Phila).

[14] Etz CD (2015). First-in-man endovascular preconditioning of the paraspinal collateral network by segmental artery coil embolization to prevent ischemic spinal cord injury. J Thorac Cardiovasc Surg.

[15] Melissano G (2010). Angio-CT imaging of the spinal cord vascularisation: a pictorial essay. Eur J Vasc Endovasc Surg.

